# Spatial and Temporal Circulation of *Babesia caballi* and *Theileria equi* in France Based on Seven Years of Serological Data

**DOI:** 10.3390/pathogens11020227

**Published:** 2022-02-09

**Authors:** Clémence Nadal, Maud Marsot, Gaël Le Metayer, Pascal Boireau, Jacques Guillot, Sarah I. Bonnet

**Affiliations:** 1 Epidemiology Unit, Laboratory for Animal Health, University Paris Est, ANSES, 94700 Paris, France; clemence.nadal@anses.fr (C.N.); maud.marsot@anses.fr (M.M.); 2 ANSES, INRAE, Ecole Nationale Vétérinaire d’Alfort, UMR BIPAR, Laboratory for Animal Health, 94700 Paris, France; 3 Parasitology Department, Ecole Nationale Vétérinaire d’Alfort, 94700 Paris, France; gael.stenuit@gmail.com (G.L.M.); jacques.guillot@oniris-nantes.fr (J.G.); 4 Veterinary Clinic of La Cère, 15800 Polminhac, France; 5 Laboratory for Animal Health, University Paris Est, ANSES, 94700 Paris, France; pascal.boireau@vet-alfort.fr; 6 Dermatology Parasitology Mycology Departement, Ecole Nationale Vétérinaire de Nantes, Oniris, 44307 Nantes, France

**Keywords:** equine piroplasmosis, *Babesia caballi*, *Theileria equi*, seroprevalence, France

## Abstract

Caused by two blood parasites, *Babesia caballi* and *Theileria equi*, equine piroplasmosis is a tick-borne disease that poses major health and economic issues for the equine industry. Our objective was to gain insight into the spatio-temporal variations of parasite circulation in France, where the disease is known to be enzootic, but has been the subject of few studies. Seroprevalence was assessed for each parasite thanks to 16,127 equine sera obtained between 1997 and 2003 from all over France and analysed through complement fixation tests. Results indicated that 13.2% (5–27% depending on the region) of horses were seropositive for *T. equi* and 9.5% (3–25%) for *B. caballi*. Regardless of the year, horses from the southern regions of France were the most affected by *B. caballi* or *T. equi* infection, while the proportion of horses having antibodies against *T. equi* increased over time. These results highlight the heterogeneity of the circulation of both piroplasms, which may be linked with ecological diversity and vector distribution. Our data provide baseline information regarding the sero-epidemiology of *B. caballi* and *T. equi* infection in horses in France, making it now possible to select regions for future studies on risk factors, and design and implement effective targeted measures against equine piroplasms.

## 1. Introduction

Equine piroplasmosis is a vector-borne disease that affects all equids (horses, donkeys, mules, zebras) and is caused by two haemoparasites of the order Piroplasmida, *Babesia caballi* and *Theileria equi* [1,2]. Both parasites are transmitted by hard tick species belonging to several genera including *Hyalomma*, *Dermacentor*, *Rhipicephalus*, *Amblyomma*, and *Haemaphysalis* [3,4]. The life cycle of both piroplasms includes asexual reproduction in the tick salivary glands (sporogony) and in horses (schizogony), as well as sexual reproduction in ticks (gamogony) [5]. However, the two parasites differ in their life cycle because, unlike *B. caballi*, *T. equi* has a pre-erythrocytic stage within equine lymphocytes and no transovarial transmission in ticks, justifying its reclassification in 1998 from genus *Babesia* to genus *Theileria* [6].

The manifestation of *B. caballi* and *T. equi* infections in equids can be symptomatic, with acute, subacute or chronic forms, or—more frequently—asymptomatic [1,2,4]. In its acute form, the disease causes severe anaemia and fever and can eventually lead to the death of the host [7,8,9]. This acute form seems more frequently observed in naïve adult horses, and less so in equids staying in enzootic areas, implying the existence of protective immunity [2,4]. The chronic form of the disease, resulting in appetite and weight loss, is mainly described in donkeys [9]. To date, the preferred treatment is imidocarb [2,10], which causes many side effects in horses [11], and does not efficiently control the disease in enzootic areas [9]. With no preventive medicine available, other than the use of acaricides to control tick infestation, equine piroplasmosis is an important equine health issue in addition to being a significant economic issue. Being considered a reportable disease by the World Organisation for Animal Health (OIE) (https://www.oie.int/en/what-we-do/animal-health-and-welfare/animal-diseases (accessed on 3 January 2022)), equine piroplasmosis affects the international trade of equids, with dramatic economic repercussions for the equine industry [9,12]. In particular, the OIE recommends that all horses be screened for *B. caballi* and *T. equi* infections prior to importation [13].

*B. caballi* and *T. equi* infections have been described worldwide, and are enzootic in many countries including several European countries bordering the Mediterranean such as France [4,14]. However, to our knowledge, only very few studies have been published on the epidemiology of these infections in France since the end of the 1990s and none of them have assessed the spatio-temporal trends in the circulation of these piroplasms [15,16,17,18,19]. The aim of the present retrospective study was thus to gain insight into the equid antibody status for both *B. caballi* and *T. equi* in France, to test statistical associations of the concomitant presence of antibodies in horses and to evaluate their spatial and temporal circulation between 1997 and 2003.

## 2. Results

### 2.1. Overall Seropositivity for Babesia caballi and Theileria equi

Out of the 16,127 sera analysed between January 1997 and December 2003, 18.5% were positive for at least one piroplasm, with 13.2% being positive for *T. equi* and 9.5% being positive for *B. caballi*; 4.1% of the sera were positive for both parasites. Among positive sera, 22.4% were positive against *T. equi* and *B. caballi*. The difference between observed seroprevalences for *T. equi* and *B. caballi* was significant (*p* < 0.05).

### 2.2. Spatial Variation of Babesia caballi and Theileria equi Seroprevalence

**Figure 1:**
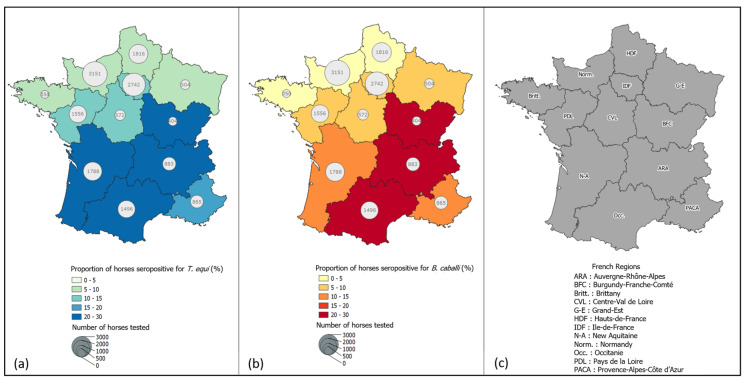
(**a**) Seroprevalence of *Theileria equi* infection among a sample of horses tested from 1997 to 2003 in France according to region; (**b**) Seroprevalence of *Babesia caballi* infection among a sample of horses tested from 1997 to 2003 in France according to region; and (**c**) Names of French regions. Black circles indicate the number of horses tested in each region.

Combining the data over the entire period (1997 to 2003), the number of tested sera varied from 350 in Brittany to 3151 in Normandy (Figure 1). Spatial analysis of seroprevalence revealed that positivity ranged from 5% in Brittany and Normandy to 27% in Auvergne-Rhône-Alpes for *T. equi* (Figure 1a), and from 3% in Brittany to 25% in Auvergne-Rhône-Alpes for *B. caballi* (Figure 1b). The highest seroprevalences for *B. caballi* and *T. equi* were observed in 5 out of 12 regions: Auvergne-Rhône-Alpes, Provence-Alpes-Côte d’Azur, Occitanie, Burgundy-Franche-Comté and New Aquitaine. On the contrary, the seven northern-most regions of France showed low seroprevalence for both piroplasms (<5%) (Figure 1).

When studying region-wise seroprevalence in horses positive for both parasites among infected animals, results showed values varying from 13.9% to 30.7% according to region, with most regions showing values between 20 and 30%.

### 2.3. Temporal Variation of Babesia caballi and Theileria equi Seroprevalence

**Figure 2:**
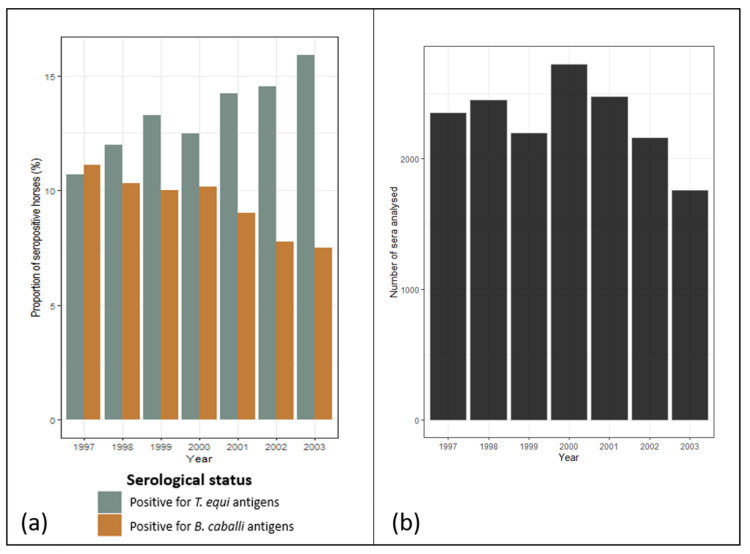
Changes in equine piroplasmosis seroprevalence rates among horses from France between 1997 and 2003. (**a**) Proportion of horses seropositive for *Theileria equi* and *Babesia caballi*; (**b**) Number of sera analysed.

Overall, across all regions, seroprevalence increased over time between 1997 and 2003 for *T. equi*, from 10.7% in 1997 to 15.9% in 2003, but decreased slightly for *B. caballi*, with a more pronounced decrease from 2000 onwards, showing values between 11.1% in 1997 and 7.5% in 2003 (Figure 2). However, the proportion of horses seropositive for at least one piroplasm remained more or less stable through the years, with values between 18.0% (in 2002) and 19.4% (in 1999).

Regarding seroprevalence over time in horses positive for both parasites among infected animals, values fluctuated from 18.0% (1997) to 26.0% (2003), revealing an increase of the proportion of horses harbouring specific antibodies against both piroplasms in France throughout the years.

### 2.4. Spatio-Temporal Variation of Babesia caballi and Theileria equi Seroprevalence

**Figure 3:**
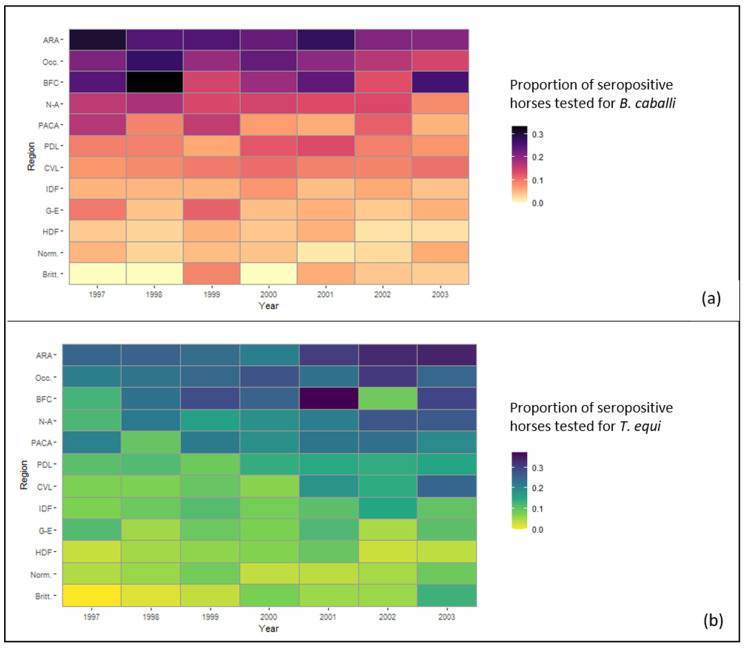
Spatio-temporal variations between 1997 and 2003 in equine piroplasmosis seroprevalence among horses from different regions of France. (**a**) Seroprevalence of *Babesia caballi*; (**b**) Seroprevalence of *Theileria equi*. ARA = Auvergne-Rhône-Alpes, Occ. = Occitanie, BFC = Burgundy-Franche-Comté, N-A = New-Aquitaine, PACA = Provence-Alpes-Côte d’Azur, PDL = Pays de la Loire, CVL = Centre-Val de Loire, IDF = Ile-de-France, G-E = Grand-Est, HDF = Hauts-de-France, Norm. = Normandy, and Britt. = Brittany.

The analysis of seropositivity for *B. caballi* in space and time showed that the general trend observed in the temporal study was mostly driven by three regions in eastern and southern France that seem particularly affected by equine piroplasmosis: Auvergne-Rhône-Alpes, Occitanie, and Burgundy-Franche-Comté (Figure 3a). Logistic regression results confirmed this observation, demonstrating that the seroprevalences for *B. caballi* in these three regions were higher (OR > 1) than that observed in the reference region (Centre-Val de Loire, with a medium rate of seropositivity against both piroplasms) (Table 1). The model also showed lower seroprevalence during the two last years of the study, when compared with the year of reference (1997), with an OR = 0.76 [0.62–0.94]_95%_ in 2002, and an OR = 0.65 [0.52–0.81]_95%_ in 2003 (Table 1).

Regarding *T. equi*, the regions exhibiting the highest percentages of seropositivity were the same as for *B. caballi* (ARA, Occ., BFC), with the addition of the New Aquitaine and Provence-Alpes-Côtes d’Azur regions in southern France (Figure 3b). The logistic model conducted on the *T. equi* serological data confirmed that these five regions had higher seroprevalences than that observed in the reference region (Table 2). It also showed a significant association of collection year (with year of reference in 1997) with the proportion of horses seropositive for *T. equi* (OR > 1 from 1998 to 2003) (Table 2).

On the contrary, regardless of the year, Brittany, Normandy, and more generally the northern third of France showed constant, but low seroprevalences of one or the other piroplasm (mostly <10%). The logistic models corroborated this observation with seroprevalences observed for both piroplasms in these regions being significantly lower (OR < 1) than that observed in the reference Centre-Val de Loire region (Table 1 and Table 2).

Finally, regarding the exploration of a potential statistical association between the concomitant presence of antibodies in sera, the association screening approach showed that the presence of a single type of antibody (anti-*T. equi* antibodies or anti-*B. caballi* antibodies) was underrepresented compared with a random association of antibodies (*p* < 0.05). On the contrary, the absence of antibodies as well as the co-occurrence of both antibodies were found to be overrepresented when compared with a random association (*p* < 0.05).

## 3. Discussion

Although equine piroplasmosis is an enzootic disease in France, no nation-wide epidemiological studies have been carried out since the 1990s to evaluate its economic or animal health consequences. To our knowledge, the present study based on serological data is the first to focus on spatio-temporal variations of *B. caballi* and *T. equi* infections in France. It focuses on the retrospective analysis of serological data obtained from blood samples of horses, including both asymptomatic equids intended for export as well as symptomatic equids, to investigate parasite transmission dynamics in France from 1997 to 2003. Our data showed strong spatial heterogeneity in the circulation of the parasites—with the southern two thirds of France being much more impacted than the northern regions—along with a significant increase of *T. equi* seroprevalence over time.

Even though antibodies persist for months or years after infection, thereby smoothing the effect of seasonality in transmission and not being adapted to detect ongoing infection in individuals, serological data represent cumulative exposure to the parasites and are widely used to monitor transmission intensity and dynamics of infectious diseases, as well as to identify high-risk groups or high-risk regions, including for equine piroplasmosis [11,20,21]. In fact, there is a direct relationship between antibody titres against *B. caballi* or *T. equi* and parasitaemia in horses [9,22], although studies have generally shown that prevalence measured based on parasite detection is lower than seroprevalence [23,24,25,26]. This difference can be attributed to the lack of sensitivity of PCR-based methods, which are designed to detect the parasites at amounts below the detection threshold (in the peripheral blood) in asymptomatic carriers or in equids having received treatment that reduces parasitaemia. However, antibody-based prevalence results also encompass the long persistence of anti-*T. equi* and anti-*B. caballi* antibodies [27].

The CFT was developed in 1945 and officially recognised as the official test for equine piroplasmosis in 1969 [28]. However, despite its good specificity, this test lacks sensitivity (47% for *T. equi* and 88% for *B. caballi*), especially in the case of latent infections [2], and it has shown occurrences of false-negative results in several studies [28,29,30]. Consequently, the indirect fluorescent antibody test (IFAT), the enzyme-linked immunosorbent assay (ELISA) and the competitive inhibition ELISA (cELISA) tests have now become the tests of choice for indirect diagnosis of *B. caballi* and *T. equi* infections and have replaced the CFT as the official tests for certifying horse movements [2,13]. Therefore, the seroprevalences measured in the present study through the CFT were likely underestimated, although analyses were all performed by the same operator throughout the entire study period, thereby limiting the reading bias usually attributed to this method.

Our results showed that 18.5% of the sera were positive for one or both piroplasms, revealing an overall increase in France compared with previous studies that were conducted on a similar sample, and also analysed with CFT in the same laboratory between 1973 and 1976 (4.3%) and between 1974 and 1988 (9%) [17,31]. Taking the parasites individually, our study reported a seroprevalence of 13.2% for *T. equi* and 9.5% for *B. caballi*, both higher than the rates measured on serological data analysed by the same operators and obtained between 1981 and 1996, with 10.2% and 7.5% of seropositive horses, respectively [32]. Since the late 1990s, to our knowledge, only one serological study has been conducted in France, at a local scale, on apparently healthy horses in the Camargue—a region in south-eastern France—and reported a seroprevalence of 58.0% against *T. equi* and 12.9% against *B. caballi* [15].

The overall higher seroprevalence against *T. equi* compared with *B. caballi* was reported in other European countries, such as Spain or Italy, following ELISA or IFAT detection tests [14,33,34]. It may be linked to the fact that lifelong *T. equi* infections are currently suspected in equids, even after treatment [1,2,9,35], with persistent low parasitaemia leading to persistent antibody titres [36]. In contrast, it is believed that clearance of *B. caballi* occurs after one to four years after infection [37], and that antibodies against *B. caballi* can persist for four to five years [35]. However, it may also reflect a higher circulation of *T. equi* than *B. caballi*. The only study conducted in France based on parasite detection focused on PCR analyses on blood samples collected from horses in the town of Marseille in south-eastern France (N = 51) and in Corsica (N = 98), a Mediterranean island, and revealed the presence of *T. equi* in Corsica and no detection of *B. caballi* [16]. Higher prevalence of *T. equi* than *B. caballi* has also been reported from Italy and Spain [38]. Regarding the co-occurrence of antibodies against *T. equi* and *B. caballi* in horses, our results highlighted a significant positive association. This may be likely explained by the fact that the two piroplasm species share vectors [3,14], with possible co-infection in ticks leading to co-infection in horses, or sequential contamination. Further studies on co-infections by both piroplasm species are needed, and experimental studies focusing on co-infections in both hosts and vectors should be conducted to explore the potential facilitation of infection between *B. caballi* and *T. equi*.

Our study also showed an overall increase in seroprevalence for *T. equi* during the course of our study, confirming what had been previously reported by Soulé and co-workers from 1981 to 1996 [18]. Meanwhile, a significant decrease in *B. caballi* seroprevalence was observed in 2002 and 2003. Considering that *T. equi* and *B. caballi* share the same vectors, this opposite dynamic as well as the higher seroprevalence and prevalence reported for *T. equi*, deserves further exploration. Unlike *B. caballi* infections, lifelong *T. equi* infections have been reported in horses [2], and imidocarb treatment is not always efficient to eliminate *T. equi*, in contrast to *B. caballi* [39,40]. In addition, infected horses are considered to be a good reservoir of *T. equi*, with possible transmission from mare to foal [41,42]. Regarding *B. caballi*, although ticks are considered a reservoir due to transovarial transmission, one study conducted on *Dermacentor nitens* reported that ticks require an alimentary reinfection on a susceptible host after one generation [43]. The likely increase in the use of acaricides and treatment of symptomatic horses with imidocarb over the past years may have led to a decrease in tick infestations in horses and better management of *B. caballi* infections, perhaps reducing *B. caballi* circulation over time, but remaining insufficient for the management of *T. equi* infections.

The serological data collected all over France also revealed high heterogeneity in the circulation of *B. caballi* and *T. equi* among regions, with greater piroplasm circulation in the southern half of the country over the years. Soulé et al. made the same observation on data collected between 1981 and 1996 [32]. This heterogeneity may be linked to more favourable conditions for *B. caballi* and *T. equi* vectors in southern France, with higher vector density and/or activity linked to a more favourable climate. For instance, vegetation cover, land use, rainfall and temperature have a direct effect on the presence and the abundance of ticks, with fluctuations according to the tick species [44,45,46]. *Dermacentor reticulatus*, *Dermacentor marginatus*, and *Rhipicephalus bursa* are the main vectors of *B. caballi* and *T. equi* in France. Thermophilic and xerophilous, *D. marginatus* is mostly found in the Mediterranean basin, but is established all over the country except in the North of France and in Brittany, where temperatures are too cold [47]. The species *D. reticulatus* is less thermophilic and xerophilous and can be found everywhere in France, but is only rarely found in Brittany and in the Mediterranean basin. Finally, *R. bursa* demonstrates a good tolerance for relatively dry microclimates, and is thus only established in the South of France, in the Mediterranean Basin [47,48]. The fact that the three vectors are mostly found in southern France, and only rarely found in—if not absent from—Brittany and the northern regions of France, may account for the distribution of seropositive horses observed in the present study. However, the heterogeneous circulation of *B. caballi* and *T. equi* in French horses may also be linked to housing conditions (outdoor vs. indoor) in the different regions, which depend on the main activity of horses reared, and directly influence horse exposure to tick bites. For example, racehorses, which are for the most part kept indoors, are mainly found in north-western France (especially in Normandy, Pays de la Loire and, to a lesser extent, Brittany), whereas draught and ride-and-drive horses, which are most often kept outside, are mainly present in southern France (Auvergne-Rhône-Alpes, Burgundy-Franche-Comté, and Occitanie) and in Brittany [49].

Unfortunately, due to a lack of individual information on horses in the dataset analysed in the present study, the clinical status of each horse was not known, making it impossible to relate seropositivity to disease expression. In the Chevrier et al. studies (1978, 1979), 8.9% of the symptomatic horses were positive, but apparently healthy animals were positive in only 2% of cases [17,31]. Similarly, Soulé et al. (1990, 1995) reported that 11.8% of horses suspected of piroplasmosis were positive, but only 2.7% of healthy animals were positive [18,19]. Our study being based on blood samples sent by practitioners, it is moreover impossible to know whether the number of sera received from each region is correlated to the number of horses residing there. To limit financial costs for the owner—especially in highly impacted areas—veterinarians often use imidocarb when piroplasmosis is suspected, even without performing any diagnostic tests, leading to a potential under-estimation of positive horses in our dataset. Therefore, although serological data are a good proxy for piroplasm circulation, the lack of knowledge on the clinical status of our studied population cumulated with the lack of reliable estimations of the horse population by region or by year in France makes it unlikely for the seroprevalences of our dataset to be reliable estimations of national seroprevalence. It can, however, be hypothesised that differences in the number of sera received from each region reflect in part the number of equids residing there. For example, Normandy is estimated to have one of the highest percentages of the national equine population, but Burgundy-Franche-Comté has a relatively low percentage of resident horses [50]. However, the very low number of sera sent from Brittany does not support this hypothesis, Brittany being one of the three most important regions of horse residency [50]. Nevertheless, considering that the AFSSA laboratory was the only one that carried out piroplasmosis tests in France for the duration of the study, our study paints a good picture of the circulation of parasites according to the French regions over time.

## 4. Materials and Methods

### 4.1. Studied Population and Sample Collection

A total of 16,127 equine serum samples were analysed using complement fixation tests (CFTs) to diagnose *B. caballi* and *T. equi* infections between January 1997 and December 2003 at the ANSES laboratory (previously called the Agence Française de Sécurité Sanitaire des Aliments (AFSSA)), Maisons-Alfort, France. Equine sera were submitted by French practitioners from all over France, either from tests carried out prior to export, or due to clinical suspicion of piroplasmosis; consequently, tests involved apparently healthy as well as symptomatic horses. For each sample, the date of blood collection and the French “department” (a French administrative division within regions) of origin were recorded and the departments were aggregated at the regional level. Unfortunately, age, sex or breed of the horses were not known. For each year and each region, the proportions of horses found positive for *B. caballi* and *T. equi* antibody detection and both parasites were evaluated.

### 4.2. Serological Detection of Babesia caballi and Theileria equi Antibodies

The blood sent by practitioners was centrifuged at 2500× *g* for 15 min to collect the serum, which was stored at 4 °C until further use. CFTs were performed within one week of sample arrival and following the protocol in Holbrook et al. [51]. Briefly, sera were inactivated at 56 °C for 30 min and used at a 1:5 dilution against both *B. caballi* and *T. equi* antigens to test the presence of antibodies specific to each parasite. Antigens were obtained from American strains from the Beltsville Laboratory (Beltsville, Maryland, USA). Each test included negative (negative sera) and positive (positive sera against the corresponding parasite) controls, as well as a negative antigen control. Each test was conducted on 96-well microplates with serial dilutions of sera from 1:5 to 1:100. The reaction was then performed in the presence of 25 µL of diluted sera, 25 µL of antigen (2 units), and 2 units of complement in 25 µL. Following incubation for 1 h at 37 °C, 50 µL of the haemolytic system was added to each well and the plates were incubated for 30 min at 37 °C. The plates were finally centrifuged for 5 min at 2000 rpm before screening. The degree of lysis was then scored from + to ++++ according to its intensity. Sera showing less than 50% lysis at a 1:5 dilution (++) were deemed positive.

### 4.3. Statistical Analysis

Overall seroprevalences of *B. caballi* and *T. equi* were estimated and compared using a χ^2^ test. The regional proportions of seropositive horses against each piroplasm species were mapped, combining the data over the entire period (1997 to 2003). The evolution of seroprevalence for *T. equi* and *B. caballi* over the seven years was then described using bar plots, across all regions. Finally, to obtain a more specific description of the distribution of both piroplasms in space and time in France, the spatio-temporal distribution of seropositive horses was represented in a tile plot, by region and by year. A multivariable logistic regression was then performed using the proportion of horses seropositive for *B. caballi* or *T. equi* per region and year as outcome, and region and year as explanatory variables. The region of reference was Centre-Val-de-Loire (CVL) (this region showed a medium rate of seropositivity against both piroplasms) and the year of reference was 1997. Results were expressed as odd ratios (OR) and 95% confidence intervals.

Finally, the proportions of horses seropositive for both piroplasms amongst horses seropositive for *B. caballi* and/or *T. equi* were evaluated along with the associated spatial and temporal variations. An association screening approach [52] was then used to test if the four combinations of antibody occurrence (presence of both antibodies—against *T. equi* and against *B. caballi*—single antibody presence—against *T. equi* or against *B. caballi*—or antibody absence) were significantly under- or over-represented compared with a random distribution. Based on the method described by Vaumourin et al., a simulated dataset was built as a presence/absence matrix with hosts in lines and antibody combinations in columns [52]. Four statistical distributions corresponding to the four combinations of antibody occurrence were obtained from 5000 simulations. A 95% confidence interval was then estimated to obtain a profile that included all the combinations simultaneously. From this profile, two quantiles, *Qinf* and *Qsup*, were inferred for each combination. Global test was based on the 95% confidence interval. When H0 (i.e., hypothesis of random distribution) was rejected, the local tests were based on the confidence intervals (*Qinf*–*Qsup*) [52].

Statistics were performed with R 3.6.1 and maps were drawn using QGIS v.3.8.3 software.

## 5. Conclusions

In conclusion, and despite a lower sensitivity than the tests used today, CFT results compiled by our laboratory between 1997 and 2003 confirmed the presence of *B. caballi* and *T. equi* across the entire French territory, with five regions from southern France being particularly affected. By shedding light on the most strongly impacted regions, studies focusing on the dynamics of piroplasm circulation can now target specific areas in the study of risk factors, which are still lacking to date. The recent establishment of equine piroplasmosis vectors previously absent from the country, such as *Hyalomma marginatum* in the South of France [53], makes it particularly important to conduct studies on the subject in the years to come. Moreover, confirming the trend observed in the years preceding our study, *T. equi* infections generally seem to have increased over the years. Given the impact of equine piroplasmosis on the French equine industry, it would be interesting to determine if this pattern still prevails today. Indeed, the present retrospective study fills a gap in our knowledge for the circulation of both *T. equi* and *B. caballi* in France and should serve as a basis for comparison of studies to be carried out today to monitor the evolution of equine piroplasmosis in this country.

## Figures and Tables

**Table 1:** Logistic regression for horse seropositivity for *Babesia caballi*, with year and region used as explanatory variables on the data collected in France between 1997 and 2003. OR, odds ratio; CI, confidence interval.

	Variable	OR	CImin	CImax
REGION	CVL	REF		
	ARA	3.28	2.39	4.58
	BFC	3.00	2.09	4.36
	Britt.	0.36	0.18	0.66
	G-E	0.69	0.43	1.09
	HDF	0.38	0.26	0.56
	IDF	0.57	0.41	0.80
	Norm.	0.37	0.26	0.52
	N-A	1.63	1.20	2.26
	Occ.	2.60	1.92	3.59
	PDL	1.05	0.76	1.48
	PACA	1.15	0.81	1.66
YEAR	1997	REF		
	1998	0.97	0.80	1.17
	1999	0.92	0.75	1.12
	2000	0.94	0.78	1.13
	2001	0.86	0.71	1.04
	2002	0.76	0.62	0.94
	2003	0.65	0.52	0.81

Variables for which the OR is significantly >1 are shown in bold, the ones for which OR < 1 are shown in italics. CVL = Centre-Val de Loire, ARA = Auvergne-Rhône-Alpes, BFC = Burgundy-Franche-Comté, Britt. = Brittany, G-E = Grand-Est, HDF = Hauts-de-France, IDF = Ile-de-France, Norm. = Normandy, N-A = New-Aquitaine, Occ. = Occitanie, PDL = Pays de la Loire, PACA = Provence-Alpes-Côte d’Azur.

**Table 2:** Logistic regression for horses seropositivity for *Theileria equi* with year and region used as explanatory variables on the data collected in France between 1997 and 2003. OR, odds ratio; CI, confidence interval.

	Variable	OR	CImin	CImax
REGION	CVL	REF		
	ARA	2.65	2.00	3.57
	BFC	2.33	1.66	3.28
	Britt.	0.40	0.23	0.66
	G-E	0.66	0.44	0.98
	HDF	0.41	0.30	0.57
	IDF	0.81	0.62	1.08
	Norm.	0.41	0.31	0.55
	N-A	1.82	1.39	2.41
	Occ.	2.41	1.84	3.19
	PDL	1.03	0.77	1.39
	PACA	1.75	1.30	2.38
YEAR	1997	REF		
	1998	1.22	1.02	1.47
	1999	1.34	1.12	1.62
	2000	1.25	1.05	1.49
	2001	1.55	1.30	1.85
	2002	1.65	1.37	1.98
	2003	1.66	1.37	2.00

Variables for which the OR is significantly >1 are shown in bold, the ones for which OR < 1 are shown in italics. CVL = Centre-Val de Loire, ARA = Auvergne-Rhône-Alpes, BFC = Burgundy-Franche-Comté, Britt. = Brittany, G-E = Grand-Est, HDF = Hauts-de-France, IDF = Ile-de-France, Norm. = Normandy, N-A = New-Aquitaine, Occ. = Occitanie, PDL = Pays de la Loire, PACA = Provence-Alpes-Côte d’Azur.
